# Supplementary material for: Clinicopathological Features of Gastric Cancer with Autoimmune Gastritis
Source: Biomedicines. 2022 Apr 12;10(4):884. doi: 10.3390/biomedicines10040884 (PMC9031450; doi:10.3390/biomedicines10040884)
Supplement: Supplementary file 1 [file biomedicines-10-00884-s001.zip › biomedicines-1644194-supplementary.pdf]

Supplementary Table 1. Patient characteristics of medications

Characteristic	APCA-positive (n = 76)	APCA-negative (n = 185)	*P* value
Medications			
Aspirin	8 (10.53)	19 (10.27)	0.951
Proton pump inhibitors	38 (50.00)	78 (42.16)	0.247
Histamine-2 receptor blocker	20 (26.32)	30 (16.22)	0.060
Statin	12 (15.79)	25 (13.51)	0.632
Nonsteroidal anti-inflammatory drugs	13 (17.11)	27 (14.59)	0.609
Angiotensin-converting enzyme blocker	8 (10.53)	17 (9.19)	0.739
Angiotensin II receptor blocker	17 (22.37)	36 (19.46)	0.596
α blocker	1 (1.32)	6 (3.24)	0.677
β blocker	10 (13.16)	21 (11.35)	0.682
Vitamin B12	13 (17.11)	22 (11.89)	0.262
Vitamin C	5 (6.58)	9 (4.86)	0.577
Metformin	3 (3.95)	8 (4.32)	0.891
